# *In vivo* spectroscopy to concurrently characterize five metabolic and vascular endpoints relevant to aggressive breast cancer

**DOI:** 10.1117/1.BIOS.1.2.025002

**Published:** 2024-07-17

**Authors:** Victoria W. D’Agostino, Riley J. Deutsch, Michelle Kwan, Enakshi D. Sunassee, Megan C. Madonna, Gregory M. Palmer, Brian T. Crouch, Nirmala Ramanujam

**Affiliations:** a Duke University, Department of Biomedical Engineering, Durham, North Carolina, United States; b Duke University, Department of Biology, Durham, North Carolina, United States; c Duke University, Department of Radiation Oncology, Durham, North Carolina, United States; d Duke University, Department of Pharmacology and Cancer Biology, Durham, North Carolina, United States

**Keywords:** tumor metabolism, optical spectroscopy, fatty acid oxidation, mitochondrial metabolism, glucose, tumor vascular environment

## Abstract

**Significance:**

Emerging evidence that aggressive breast tumors rely on various substrates including lipids and glucose to proliferate and recur necessitates the development of tools to track multiple metabolic and vascular endpoints concurrently *in vivo*.

**Aim:**

Our quantitative spectroscopy technique provides time-matched measurements of the three major axes of breast cancer metabolism as well as tissue vascular properties *in vivo*.

**Approach:**

We leverage exogenous fluorophores to quantify oxidative phosphorylation, glucose uptake, and fatty acid oxidation, and endogenous contrast for measurements of hemoglobin and oxygen saturation. An inverse Monte Carlo algorithm corrects for aberrations resulting from tissue optical properties, allowing the unmixing of spectrally overlapping fluorophores.

**Results:**

Implementation of our inverse Monte Carlo resulted in a linear relationship of fluorophore intensity with concentration ($R^{2}<0.99$) in tissue-mimicking phantom validation studies. We next sequenced fluorophore delivery to faithfully recapitulate independent measurement of each fluorophore. The ratio of Bodipy FL C16/2-NBDG administered to a single animal is not different from that in paired animals receiving individual fluorophores (p=n.s.). Clustering of five variables was effective in distinguishing tumor from mammary tissue (sensitivity = 0.75, specificity = 0.83, and accuracy = 0.79).

**Conclusions:**

Our system can measure major axes of metabolism and associated vascular endpoints, allowing for future study of tumor metabolic flexibility.

Statement of Discovery We describe a novel method leveraging quantitative fluorescence spectroscopy to characterize oxidative phosphorylation, glucose uptake, fatty acid uptake, total hemoglobin, and oxygen saturation concurrently in healthy and tumor-bearing *in vivo* murine tissue.

## 1 Introduction

The ability of cancer cells to utilize multiple metabolic pathways to produce ATP is foundational to their ability to rapidly proliferate, resist therapy, and progress to metastatic disease.^1^^,^^2^ Myriad studies have shown that cancers procure energy not only through aerobic glycolysis but also through oxidative phosphorylation (OXPHOS) and rely on substrates such as glucose, lipids, and amino acids.^2^^–^^4^ Many have observed the ability of tumors to switch between metabolic pathways for enhanced survival.^3^^,^^5^^,^^6^ Furthermore, lack of organized vasculature in rapidly proliferating tumors also results in tumor cells existing in various states of nutrient and oxygen deprivation.^4^ Oxygen-depleted environments, created as tumor biomass is rapidly accumulated, result in the activation of hypoxia-inducible factors.^4^ These transcription factors mediate responses, such as triggering angiogenesis and metabolic shifts, both hallmarks of cancer and drivers of cancer aggression.^4^ Further, the gradients in nutrient availability and metabolic pathway utilization are therefore highly impacted by the unbalanced distribution of blood vessels.^4^ Clinical management of breast cancer relies on metabolic imaging of [F18] fluoro-2-deoxyglucose positron emission tomography ([F18]FDG PET) for staging, monitoring of tumor response, and the detection of recurrent disease.^5^^,^^6^ [F18]FDG PET imaging is based on the well-studied principle that cancerous tissues rely on the increased uptake of glucose to fuel aerobic glycolysis.^6^^,^^7^ However, this imaging modality focuses on a single metabolic endpoint, glucose. Cancerous tissues also have the capacity to be metabolically flexible and can rely on both oxidative metabolism and glycolysis to meet their energy demands.^4^

Recent studies by our group and others have highlighted the ability of aggressive breast cancer to reprogram its metabolic preferences to achieve treatment resistance and recurrence.^8^ In one study, preclinical observation of increased fatty acid metabolism in residual disease was mirrored in clinical samples from cells surviving neoadjuvant treatment in human breast cancer patients.^9^ In another study, metastatic disease from 161 breast cancer patients were characterized by an oxidative phenotype.^1^ The significance of fatty acid uptake and oxidative metabolism has also been observed in genetically engineered mouse models of breast cancer. Primary tumors in a recurrent model of HER2+ breast cancer displayed a shift from glycolysis to OXPHOS over the course of regression, dormancy, and recurrence.^3^^,^^8^^–^^12^ The increased reliance of an MYC oncogene expressing model of Triple Negative Breast Cancer (TNBC) on fatty acids was reversed with drugs targeting lipid metabolism.^8^^,^^13^ Further, our group has reported a significant positive correlation in metabolic and vascular endpoints in murine models of TNBC.^14^ These relationships were observed in aggressively metastatic murine TNBC tumors but not in their micro- or non-metastatic counterparts.^15^ These collective studies point to the metabolic flexibility of aggressive tumors and the importance of measuring the major players that contribute to this major hallmark of cancer survival.

Our group has extensively validated three exogenous contrast agents to report on relevant metabolic axes of breast cancer.^3^^,^^8^^,^^11^^,^^14^^–^^20^ Tetramethylrhodamine ethyl ester, a fluorescent cation attracted to the negative charge created by metabolically active mitochondria, has been validated as a surrogate for OXPHOS.^16^^,^^21^ The fluorescent glucose analog 2-(N-(7-nitrobenz-2-oxa-1,3-diazol-4-yl) amino)-2-deoxyglucose (2-NBDG, reports on the uptake of glucose) along with TMRE allows for near-simultaneous monitoring of both glucose uptake and OXPHOS.^14^^–^^19^ A temporally spaced injection scheme of 20 min between TMRE (first agent) and 2-NBDG (second agent) mitigates biological crosstalk.^14^^,^^19^ Further, we have demonstrated that TMRE and Bodipy FL C16 (reports on the uptake of fatty acids) can be combined in a simultaneous injection scheme without optical or biological crosstalk.^20^

In this work, we sought to develop a methodology to quantify the key nutrients that drive OXPHOS—fatty acids and glucose—by minimizing crosstalk between Bodipy FL C16 and 2-NBDG. First, we optimized the selection of excitation wavelengths to minimize optical crosstalk between the overlapping spectra of Bodipy FL C16 and 2-NBDG. Second, we minimized biological crosstalk by timing the sequencing of Bodipy FL C16 within the current injection scheme for the TMRE, 2-NBDG pair. Third, we demonstrated that independent measurements of each fluorophore faithfully captured OXPHOS, glucose uptake, and fatty acid oxidation. These collective endpoints along with spectroscopic measurements of total hemoglobin ([THb]) and oxygen saturation (${\mathrm{SO}}_{2}$) allow for the investigation of both metabolic substrates and vascular endpoints, all of which affect OXPHOS. We observed that the relationship between the ratio of TMRE and the substrates (TMRE/(2-NBDG + Bodipy FL C16)) was statistically higher in tumor compared to non-tumor bearing mammary tissues. Though there were no statistical differences in [THb] or ${\mathrm{SO}}_{2}$ between tumor and non-tumor tissues, clustering analysis of all five variables was more effective in discriminating the tissue types compared to the metabolic endpoints alone.

The capability to measure the major metabolic axes and the associated vascular endpoints plays an important role in cancer therapeutics.^22^^,^^23^ It allows for the study of tumor metabolic flexibility and plasticity, features that are inextricably linked to treatment resistance, recurrence, and metastasis. Further, it can identify critical windows of opportunity for detailed analyses with metabolomics (bulk tissue analysis), RNA sequencing, and immunohistochemistry (IHC), methods that are highly reliant on the time points at which the tissues are harvested. The same technology can inform the selection of single or combination therapies by measuring the efficacy of these inhibitors in preventing metabolic reprogramming.

## 2 Methods

### 2.1 Ethics Statement

All animal work was carried out in accordance with the recommendations in the Guide for the Care and Use of Laboratory Animals of the National Institutes of Health. The protocol was approved by the Duke University Institutional Animal Care and Use Committee (protocol number A038-21-02). All mice were housed in an on-site housing facility with *ad libitum* access to food and water with standard light/dark cycles. Mice were monitored daily for tumor growth by inspection and caliper measurement and were sacrificed as per ethical guidelines. All experiments were performed under isoflurane gas anesthesia, and all efforts were made to minimize suffering.

### 2.2 Cell Culture

All cell lines used herein were 4T1s purchased from the American Type Culture Collection (ATCC) and subjected to mycoplasma testing (ATCC number CRL-2539). 4T1 cells were cultured in RPMI (Gibco, Montgomery County, Maryland, 11875093) supplemented with 10% fetal bovine serum (Gibco, Montgomery County, Maryland, A3160501) and 1% antibiotics (penicillin/streptomycin, Gibco, Montgomery County, Maryland, 15140122). Cells were incubated at 37°C with 5% ${\mathrm{CO}}_{2}$ and 95% relative humidity and passaged at ∼80% confluency twice before injection into animals.

### 2.3 Fluorescent Metabolic Indicators

Fluorescent cation TMRE (Thermo Fisher Scientific, Waltham, Massachusetts) was used to measure mitochondrial membrane potential. It accumulates inside of cells due to its electrostatic attraction to the proton gradient generated by the active mitochondria, causing a measurable increase in fluorescence intensity.^16^^,^^17^^,^^21^ Our group has conducted robust validation of TMRE as a surrogate for OXPHOS; we have previously measured TMRE signal in response to a chemical perturbation CCCP, a compound that dissipates mitochondrial membrane potential.^16^ We observed the expected response in both tumor-bearing and non-tumor-bearing tissues: a significant decrease in TMRE signal after perturbation with CCCP.^16^ For *in vivo* studies, TMRE was diluted to a final concentration of 25  μM in sterile phosphate buffered serum (PBS, Gibco, Montgomery County, Maryland, 10010023); this dose was optimized in prior work such that the tissue-level concentration is 50 nM to prevent quenching and electron transport chain disruption.^16^^,^^17^^,^^21^^,^^24^^–^^26^

2-NBDG (Thermo Fisher Scientific, Waltham, Massachusetts) is a fluorescent analog to glucose. It is transported into the cell via GLUT, the same membrane transport protein that transports glucose into the cell and accumulates as it cannot be metabolized through glycolysis. It produces a measurable fluorescence intensity change reflecting glucose uptake.^25^ Thus, 2-NBDG is a surrogate for glucose uptake; when coupled with TMRE in a single animal, 2-NBDG provides information relating to glycolysis and/or oxidative phosphorylation of glucose molecules. For *in vivo* studies, 2-NBDG was diluted to a final concentration of 6 mM in sterile PBS (Gibco, Montgomery County, Maryland, 10010023), a dose optimized in a prior publication.^17^

Bodipy FL C16 (4,4-difluoro-5,7-dimethyl-4-bora-3a,4a-diaza-s-indacene-3-hexadecanoic acid, Thermo Fisher Scientific, Waltham, Massachusetts) is a fluorescently labeled palmitate molecule that is taken up similarly to palmitate but not immediately metabolized due to the fluorescent label on the 16th carbon of the palmitate.^11^ Bodipy fluorescence was collected at its emission peak of 512 nm, which corresponds to its protein-bound state, the default condition *in vivo*.^27^ For *in vivo* studies, Bodipy FL C16 was diluted to a final concentration of 200  μM in sterile PBS (Gibco, Montgomery County, Maryland, 10010023), a dose optimized previously to achieve tissue-level concentrations that avoid self-quenching.^11^^,^^27^

### 2.4 Optical Spectroscopy Measurements

Optical measurements for all studies were collected using a previously reported optical spectroscopy system and optical fiber-based probe.^19^ The spectroscopy system consists of a 450-W Xenon lamp, a monochromator, a spectrograph, and a 2D CCD camera (Jobin Yvon Horiba, Edison, New Jersey). The probe consists of 19 fibers for excitation illumination and 18 fibers for emission detection (RoMack Inc., Irving, Texas). Each fiber has a numerical aperture of 0.22 and an estimated sensing depth of 1.5 mm. To account for day-to-day variations in the system, reflectance and fluorescence spectra were calibrated to a 20% reflectance standard (Spectralon Labsphere Inc., North Sutton, New Hampshire) and a fluorescence standard (USF 210-010, Spectralon Labsphere Inc., North Sutton, New Hampshire), respectively. Diffuse reflectance measurements were collected by illuminating the tissue with broadband white light and scanning the emission monochromators across the wavelength range of interest (350 to 700 nm). Fluorescence measurements were collected by fixing the source monochromator to provide the required excitation wavelength while scanning the detection monochromator over the desired spectral range. All measurements were acquired in a dark room. The system was allowed to warm up adequately (30 min or more) before collecting any measurements. Reflectance spectra were acquired from 420 to 760 nm (acquisition time: 0.003 s). Bodipy FL C16 and 2-NBDG fluorescence spectra were acquired from 505 to 635 nm (acquisition time for *in vivo* measurements: 1 s; acquisition time for phantom measurements: 0.1 s) using excitation at 488 nm. TMRE fluorescence spectra were acquired from 575 to 705 nm (acquisition time: 5 s) using excitation at 555 nm. Background spectra were collected for both phantoms and animals before the addition of fluorophore. All spectra and peak intensity values shown have been corrected using the inverse Monte Carlo and spectral unmixing, described below, unless otherwise noted.

### 2.5 Inverse Monte Carlo Model for Reflectance and Fluorescence

A scalable inverse Monte Carlo model was used to extract optical properties and intrinsic fluorescence signal from both tissue and liquid phantoms. Measured reflectance and fluorescence spectra were input into the model to correct for effects of tissue optical properties, namely absorption and scattering. Previous work has validated the inverse Monte Carlo model’s ability to extract both optical properties and intrinsic fluorescence signal from liquid phantoms and tissue.^28^^–^^32^ To correct the measured fluorescence spectrum for effects of tissue optical properties, it is necessary to know the optical absorption and scattering properties of the medium. The inverse Monte Carlo first extracts the absorption coefficient ($\mu_{a}$) and reduced scattering coefficient (${\mu_{s}}^{\prime }$) from a diffuse reflectance spectrum. These extracted optical properties are then used by the inverse Monte Carlo fluorescence model to correct for the effects of scattering and absorption on the measured fluorescence spectrum to provide the intrinsic fluorescence. A measured reference phantom with known optical properties is used to generate the calibration factor to appropriately scale the outputs of the inverse Monte Carlo algorithm. The reference phantom was selected to minimize the error of extracting both absorption and scattering as previously described.^29^

### 2.6 Spectral Unmixing of Combined 2-NBDG and Bodipy FL C16 Spectra

**Fig. 1:**
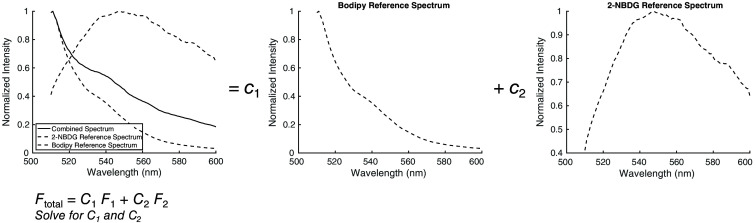
The linear unmixing strategy used to separate Bodipy FL C16 and 2-NBDG spectra. Representative examples of the combined spectrum (solid line) and the individual (reference) spectrum for each fluorophore (dashed lines). The equation used is $F_{\text{total}}=C_{1}F_{1}+C_{2}F_{2}$.

To distinguish the spectra of Bodipy FL C16 and 2-NBDG when both fluorophores are present in either tissue or liquid phantom, a linear unmixing strategy was used as shown in Fig. 1. The combined spectrum can be assumed to be a linear combination of each individual spectrum.

Thus, after a measured spectrum was corrected for tissue optical properties using the previously described inverse Monte Carlo algorithm, it was input into a MATLAB (Mathworks, Natick, Massachusetts) script to generate a coefficient matrix, which would minimize the error of Eq. (1):

F=(a(1)*Bodipy)+(a(2)*NBDG), (1)

where “Bodipy” is a reference spectrum for Bodipy FL C16 and “NBDG” is a reference spectrum for 2-NBDG. Reference spectra were generated by measuring the single fluorophore using the same experimental parameters (excitation and emission wavelengths, integration time, and composition of the phantom including concentration of scatterer and absorber). a(1) and a(2) are the coefficients for Bodipy FL C16 and 2-NBDG, respectively, which are solved for using the MATLAB (Mathworks, Natick, Massachusetts) function lsqcurvefit. The resulting solution to Eq. (1) is a matrix containing the coefficients to the Bodipy FL C16 and 2-NBDG reference spectra, representing their contribution to the mixed spectrum. After generating the coefficient matrix for each sample, any further analysis utilizes those coefficients multiplied by the reference spectrum.

### 2.7 Unmixing Absorption Spectra of Non-Negligible Absorbers 2-NBDG and Hemoglobin

Similarly, to distinguish the absorption spectrum of hemoglobin from that of fluorophore 2-NBDG when both absorbers are present in either tissue or liquid phantom, a linear least-squares fit algorithm was used. After absorption spectra were extracted using the previously described inverse Monte Carlo algorithm, the extracted absorption spectra were input into a MATLAB (Mathworks, Natick, Massachusetts) script to generate a coefficient matrix, which would minimize the error in Eq. (2):

$$F=(a(1)*{\mathrm{HbO}}_{2})+(a(2)*\mathrm{Hb})+(a(3)*\mathrm{NBDG}),$$ (2)

where “${\mathrm{HbO}}_{2}$” is a reference absorption spectrum for oxy-hemoglobin, “Hb” is a reference absorption spectrum for deoxy-hemoglobin, and “NBDG” is a reference absorption spectrum for 2-NBDG. The reference spectrum for 2-NBDG was generated by measuring the single fluorophore using a gold standard spectrophotometer (Cary 300, Varian, Inc., Palo Alto, California) and the oxy- and deoxy-hemoglobin reference absorption spectra from a publicly available database.^33^ a(1), a(2), and a(3) are the coefficients for oxy-hemoglobin, deoxy-hemoglobin, and 2-NBDG, respectively, which are solved for using the MATLAB (Mathworks, Natick, Massachusetts) function lsqcurvefit. The same unmixing process and the same reference absorption spectra were used across all experiments. After generating the coefficient matrix for each sample, any further analysis utilizes coefficients multiplied by the reference spectrum.

### 2.8 Liquid Phantoms

Three sets of liquid phantoms were created to demonstrate that (i) the inverse Monte Carlo algorithm can reliably extract the intrinsic Bodipy FL C16 and 2-NBDG signal from phantoms with different optical properties, (ii) optical crosstalk between Bodipy FL C16 and 2-NBDG when combined in a scattering solution containing a constant level of TMRE can be mitigated using spectral unmixing, (iii) Bodipy FL C16, 2-NBDG, and tissue optical properties can be reliably extracted from optical spectra using the inverse Monte Carlo algorithm across a biologically relevant range of concentrations in tissue-mimicking phantoms.

Liquid phantoms with tissue-mimicking properties were prepared using varying absorber, scatterer, and fluorophore concentrations. Hemoglobin (H0267, Sigma-Aldrich Co., St. Louis, Missouri) was used as the non-fluorescent absorber, and 1  μm monodisperse polystyrene spheres (1  μm diameter, Catalog No. 07310, Polysciences, Warrington, Pennsylvania) were used as the scatterer. When protein-bound, the emission peak of Bodipy FL C16 is 512 nm as previously characterized; the base of all phantoms was 0.2  g/mL of bovine serum albumin (A7906, Sigma-Aldrich Co., St. Louis, Missouri) in phosphate-buffered saline (PBS/BSA) to allow for protein binding (the default state *in vivo*).^27^ Mixing known volumes of stock hemoglobin and microsphere suspensions in PBS/BSA with stock fluorophore solution allowed accurate control of the final absorption, scattering, and fluorescence properties in each phantom. Fluorophore concentrations are based on biologically relevant fluorophore concentrations established in previous *in vivo* microscopy studies.^11^^,^^14^ The absorption spectra of the stock hemoglobin were measured using a spectrophotometer (Cary 300, Varian, Inc., Palo Alto, California) and were used to determine the final absorption of the phantom, whereas the values of ${\mu_{s}}^{\prime }$ in the phantoms were calculated from the Mie theory for spherical particles.^34^ Reflectance and fluorescence measurements from the phantoms were obtained by placing the optical probe just beneath the surface of the liquid with the phantom being well mixed by repeated pipetting before each measurement. Data reported are single measurements unless noted otherwise.

#### 2.8.1 Extracting intrinsic Bodipy FL C16 and 2-NBDG signal from phantoms with different optical properties using the inverse Monte Carlo algorithm

Raw intensity measurements of fluorophore in solution are affected by the ${\mu_{s}}^{\prime }$ of the solution; our group has previously shown this effect to be corrected by an inverse Monte Carlo model in a single fluorophore preparation of Bodipy FL C16.^20^ Here, when combining Bodipy FL C16 and 2-NBDG for the first time, we sought to demonstrate the ability of the inverse Monte Carlo algorithm to correct for the effects of varying ${\mu_{s}}^{\prime }$ on the combined Bodipy FL C16 and 2-NBDG spectrum. To do so, we constructed two pairs of turbid phantoms and one pair of non-turbid phantoms as shown in Table 1. Each pair of turbid phantoms contained Bodipy FL C16 at a low (200 nM) and high (400 nM) concentration along with a fixed concentration of 2-NBDG (0.5  μM), a fixed $\mu_{a}$ ($0.3\;{\mathrm{cm}}^{-1}$), and scatterer. One phantom pair had a high level of scatterer (${\mu_{s}}^{\prime }=20\;{\mathrm{cm}}^{-1}$) and the other contained a low level of scatterer (${\mu_{s}}^{\prime }=10\;{\mathrm{cm}}^{-1}$). Non-turbid phantoms were constructed containing the above concentrations of Bodipy FL C16 and 2-NBDG without scatterer or absorber as a gold standard. To appropriately compare non-turbid and turbid phantoms, a scaling factor was applied to all non-turbid spectra based on the ratio of the peak of the phantom containing 200 nM Bodipy FL C16 and ${\mu_{s}}^{\prime }=20\;{\mathrm{cm}}^{-1}$ to the peak of the non-turbid phantom containing 200 nM Bodipy FL C16 (a method established previously).^20^

**Table 1:** A series of turbid and non-turbid liquid phantoms containing Bodipy FL C16, 2-NBDG, hemoglobin, and microspheres at varying concentrations were prepared. The $\mu_{a}$ (${\mathrm{cm}}^{-1}$), ${\mu_{s}}^{\prime }$ (${\mathrm{cm}}^{-1}$), and fluorophore concentrations for each phantom are shown.

	Bodipy FL C16 (nM)	${\mu_{s}}^{\prime }$ (${\mathrm{cm}}^{-1}$)	2-NBDG (nM)	$\mu_{a}$ (${\mathrm{cm}}^{-1}$)
Turbid phantoms	200	20	500	0.3
	400	20	500	0.3
	200	10	500	0.3
	400	10	500	0.3
Non-turbid phantoms	200	0	500	0
	400	0	500	0

#### 2.8.2 Mitigating optical crosstalk between Bodipy FL C16 and 2-NBDG using spectral unmixing

To validate that optical crosstalk due to the overlapping excitation and emission spectra of 2-NBDG and Bodipy FL C16 that can be mitigated using spectral unmixing, four sets of liquid phantoms were constructed containing either (i) Bodipy FL C16 at concentrations 0, 0.2, 0.4, 0.6, 0.8, and 1  μM; (ii) 2-NBDG at concentrations 0, 2, 4, 6, 8, and 10  μM; (iii) Bodipy FL C16 at the same concentration range with a fixed concentration of 8  μM 2-NBDG and a fixed concentration of 9 nM TMRE; or (iv) 2-NBDG at the same concentration range with a fixed concentration of 0.8  μM Bodipy FL C16 and a fixed concentration of 9 nM TMRE. These phantoms contained fluorophore and polystyrene microspheres as the scatterer (${\mu_{s}}^{\prime }=10\;{\mathrm{cm}}^{-1}$) as shown in Table 2. No absorber was included. This was repeated in triplicate, and peak intensity is shown as the averaged peak intensity with error bars being standard error of the mean. Representative spectra are shown from one experimental repeat.

**Table 2:** A series of turbid liquid phantoms containing Bodipy FL C16, 2-NBDG, and TMRE at varying concentrations with a constant concentration of scatterer were prepared. The $\mu_{a}$ (${\mathrm{cm}}^{-1}$), ${\mu_{s}}^{\prime }$ (${\mathrm{cm}}^{-1}$), and fluorophore concentrations for each phantom are shown.

Phantom	2-NBDG (μM)	Bodipy FL C16 (μM)	TMRE (nM)	${\mu_{s}}^{\prime }$ (${\mathrm{cm}}^{-1}$)	$\mu_{a}$ (${\mathrm{cm}}^{-1}$)
Individual Bodipy FL C16 phantoms					
Background	0	0	0	10	0
1	0	0.2	0	10	0
2	0	0.4	0	10	0
3	0	0.6	0	10	0
4	0	0.8	0	10	0
5	0	1.0	0	10	0
Individual 2-NBDG phantoms					
Background	0	0	0	10	0
1	2	0	0	10	0
2	4	0	0	10	0
3	6	0	0	10	0
4	8	0	0	10	0
5	10	0	0	10	0
Mixed Bodipy FL C16 phantoms					
Background	8	0	9	10	0
1	8	0.2	9	10	0
2	8	0.4	9	10	0
3	8	0.6	9	10	0
4	8	0.8	9	10	0
5	8	1.0	9	10	0
Mixed 2-NBDG phantoms					
Background	0	0.8	9	10	0
1	2	0.8	9	10	0
2	4	0.8	9	10	0
3	6	0.8	9	10	0
4	8	0.8	9	10	0
5	10	0.8	9	10	0

#### 2.8.3 Extracting Bodipy FL C16, 2-NBDG, and tissue optical properties from optical spectra using the inverse Monte Carlo algorithm across a biologically relevant range of concentrations in a tissue-mimicking phantom

Finally, a set of tissue-mimicking phantoms was created to emulate biologically relevant ranges of both scatterer and absorber to demonstrate that reflectance and fluorescence measurements made at the same time with a single instrument can quantify four endpoints: fluorescence intensities of Bodipy FL C16 and 2-NBDG, $\mu_{a}$ (${\mathrm{cm}}^{-1}$), and $\mu_{s}$ (${\mathrm{cm}}^{-1}$). A series of 10 phantoms was constructed as shown in Table 3. ${\mu_{s}}^{\prime }$ decreases from 15.8 to $5.64\;{\mathrm{cm}}^{-1}$, $\mu_{a}$ increases from 15.09 to $53.89\;{\mathrm{cm}}^{-1}$, Bodipy FL C16 concentration increases from 0 to 998.6 nM, and 2-NBDG concentration decreases from 10 to 3.57  μM. Values reported are average ${\mu_{s}}^{\prime }$ and $\mu_{a}$ across wavelengths 450 to 600 nm, Bodipy FL C16 at its emission peak (512 nm), and 2-NBDG at its emission peak (545 nm). The diffuse reflectance and fluorescence at 488 nm were measured for each phantom. Expected hemoglobin concentration is calculated using stock absorbance measured by spectrophotometer (Cary 300, Varian, Inc., Palo Alto, California) while extracted hemoglobin concentration is calculated using $\mu_{a}$ extracted by inverse Monte Carlo.

**Table 3:** A series of 10 tissue-mimicking liquid phantoms containing Bodipy FL C16, 2-NBDG, hemoglobin, and microspheres at varying concentrations were prepared. The mean $\mu_{a}$ (${\mathrm{cm}}^{-1}$), mean ${\mu_{s}}^{\prime }$ (${\mathrm{cm}}^{-1}$), hemoglobin concentration (μM), and fluorophore concentrations for each phantom are shown.

Phantom	${\mu_{s}}^{\prime }$^a^ (${\mathrm{cm}}^{-1}$)	$\mu_{a}$^b^ (${\mathrm{cm}}^{-1}$)	[Hb] (μM)	[2-NBDG] (μM)	[Bodipy FL C16] (nM)
Background	15.80	0.14	15.09	0.00	0.00
1	15.80	0.14	15.09	10.00	0.00
2	13.16	0.24	25.15	8.33	259.00
3	11.28	0.30	32.33	7.14	444.00
4	9.87	0.35	37.72	6.25	582.00
5	8.78	0.39	41.81	5.56	690.00
6	7.90	0.42	45.26	5.00	776.50
7	7.81	0.45	48.01	4.55	847.00
8	6.58	0.47	50.29	4.17	906.00
9	6.08	0.49	52.23	3.85	955.90
10	5.64	0.50	53.89	3.57	998.60

a Mean ${\mu_{s}}^{\prime }$

b Mean $\mu_{a}$

### 2.9 *In Vivo* Murine Breast Cancer Model Studies

As Bodipy FL C16 and 2-NBDG were the two fluorophores that had yet to be combined *in vivo*, the first murine experiment was designed to validate that Bodipy FL C16 and 2-NBDG could be combined and measured without biological crosstalk. Once that was proven, we demonstrated our concurrent injection scheme in a murine TNBC model (4T1, ATCC number CRL-2539) in the mammary fat pad (orthotopic) and in healthy mammary tissue.

Female BALB/c mice (Charles River Laboratories, Raleigh, North Carolina) weighing 25 to 30 g at 5 to 6 weeks of age were used in all animal studies. All animal experiments were conducted during the day, and mice were fasted for at least 2 h prior to optical measurements (with *ad libitum* access to water). Fasting ensured glucose in the body did not compete with fluorophore uptake and signal contrast from the tumor compared to normal tissue.^28^ Fasting was confirmed by measuring blood glucose levels with glucose test strips (Abbott, Alameda, California). To collect reflectance and fluorescence spectra from animals, the probe was placed to gently contact the tumor or healthy mammary fat pad, without compressing the surface or leaving an air gap, and stabilized in the same place for the duration of imaging (between 60 and 80 min) using a custom holder. Animals were anesthetized via isoflurane inhalation (1% to 2% isoflurane gas mixed with oxygen) throughout the course of the optical measurements.

For all tumor studies, 4T1 (ATCC number CRL-2539) murine breast cancer cell lines were used to grow orthotopic mammary tumors. At 5 weeks of age, each mouse received a 100  μL subcutaneous injection of 30,000 cells in the fourth right mammary fat pad. Tumors were monitored every other day and allowed to grow to a volume of $150\;{\mathrm{mm}}^{3}$, calculated by $(\text{length}\times {\text{width}}^{2})/2$, with length recorded as the longest axis and width the shortest. $150\;{\mathrm{mm}}^{3}$ is a palpable volume that provides a sufficient surface for spectroscopy, without resulting in ulcerations.

At the start of each study, mice were randomized across cages to minimize batch effects. All fluorophore injections were done retro-orbitally and were 100  μL each. All fluorophores were diluted in PBS (Gibco, Montgomery County, Maryland, 10010023) for injections. At each time point, three measurements were collected: TMRE fluorescence at 555 nm excitation, Bodipy FL C16 and 2-NBDG fluorescence at 488 nm excitation, and diffuse reflectance spectra. The inverse Monte Carlo algorithm extracted intrinsic Bodipy FL C16 fluorescence, intrinsic 2-NBDG fluorescence, intrinsic TMRE fluorescence, ${\mu_{s}}^{\prime }$ and $\mu_{a}$, and ${\mathrm{SO}}_{2}$ and [THb] from $\mu_{a}$. Spectral unmixing was used to unmix Bodipy FL C16 and 2-NBDG as described above.

To demonstrate that there is no biological crosstalk between Bodipy FL C16 and 2-NBDG, two fluorophores which have never been combined *in vivo* previously, we sought to show that temporally spaced measurements of Bodipy FL C16 and 2-NBDG (staggered injection) produced equivalent results to measurements of either Bodipy FL C16 or 2-NBDG alone in orthotopic murine 4T1 solid tumors and healthy mammary fat pad tissue.

To investigate biological crosstalk between Bodipy FL C16 and 2-NBDG in healthy mammary tissue, a total of 24 healthy animals were divided into three groups of eight. Mice were randomized into the following study groups, each with a cohort size of n=8: cohort i) received an injection of Bodipy FL C16 (200  μM), cohort ii) received an injection of 2-NBDG (6 mM), and cohort iii) received an injection of Bodipy FL C16 (200  μM) first followed by an injection of 2-NBDG (6 mM) after 20 min. One mouse from cohort iii) passed away, so the cohort size reported is n=7.

To investigate biological crosstalk between Bodipy FL C16 and 2-NBDG in orthotopic 4T1 bearing mice, a total of 18 4T1 tumor-bearing mice were divided into three groups of six. Mice were randomized into the following study groups, each with a cohort size of n=6: cohort i) received an injection of Bodipy FL C16 (200  μM), cohort ii) received an injection of 2-NBDG (6 mM), and cohort iii) received an injection of Bodipy FL C16 (200  μM) first followed by an injection of 2-NBDG (6 mM) after 20 min.

We next sought to investigate a concurrent injection scheme of all three fluorophores compared to the gold standard injection scheme temporally spaced over three days. In both normal mammary fat pad tissue and orthotopic 4T1 tumors, a total of 12 mice were divided into two groups of six per tissue type. Mice were randomized into the following study groups, each with a cohort size of n=6: cohort i) a multi-day injection scheme with TMRE (75  μM) injected first followed by 2-NBDG (6 mM) injected after 20 min, with Bodipy FL C16 (200  μM) injected and measured 2 days later and ii) a concurrent injection scheme with a dual injection of mixed Bodipy FL C16 (200  μM) and TMRE (75  μM) injected first followed by another injection of 2-NBDG (6 mM) injected after 20 min. 60-min post-injection timepoints for Bodipy FL C16, 2-NBDG, and TMRE are referred to as ${\text{Bodipy}}_{60}$, $2\text{-}{\mathrm{NBDG}}_{60}$, and ${\mathrm{TMRE}}_{60}$, respectively.

### 2.10 Data Analysis

All $R^{2}$ values reported are the lines of best fit with the y-intercept forced to (0,0). Unless otherwise noted, all spectral data shown are corrected by our previously developed inverse Monte Carlo model.^28^^–^^32^ All Bodipy FL C16 and 2-NBDG spectral data shown are spectrally unmixed via the above methods unless otherwise noted. Vascular endpoints are calculated using the background reflectance spectra (before injection of fluorophores).

Comparisons were made using the Wilcoxon rank-sum test. A p-value of 0.05 or less was considered statistically significant; * denotes a p value <0.05, ** denotes a p value <0.01, and *** denotes a p value <0.001. MATLAB (Mathworks, Natick, Massachusetts) was used to perform all statistical analyses and calculations unless otherwise noted.

Before calculating metabolic ratios (${\mathrm{TMRE}}_{60}/({\text{Bodipy}}_{60}+2\text{-}{\mathrm{NBDG}}_{60})$, ${\mathrm{TMRE}}_{60}/{\text{Bodipy}}_{60}$, ${\text{Bodipy}}_{60}/2\text{-}{\mathrm{NBDG}}_{60}$ and ${\mathrm{TMRE}}_{60}/2\text{-}{\mathrm{NBDG}}_{60}$), data for each endpoint were normalized by dividing each point by the maximum measured value of that endpoint.

Principal component analysis (PCA) was used for dimensionality reduction. PCA was performed on different combinations of normalized ${\text{Bodipy}}_{60}$, ${\mathrm{TMRE}}_{60}$, $2\text{-}{\mathrm{NBDG}}_{60}$, ${\mathrm{SO}}_{2}$, and [THb] from both the concurrent and delayed injection schemes. As a secondary investigation, Uniform Manifold Approximation and Projection (UMAP) was used for dimensionality reduction^35^ of different combinations of ${\text{Bodipy}}_{60}$, ${\mathrm{TMRE}}_{60}$, $2\text{-}{\mathrm{NBDG}}_{60}$, ${\mathrm{SO}}_{2}$, and [THb] from both the concurrent and delayed injection schemes (Fig. S2 in the Supplementary Material).

Both the metabolic and vascular ratios and the PCA output were clustered using a k-means clustering algorithm (built-in to MATLAB, Mathworks, Natick, Massachusetts) for both mammary and tumor tissue data. To quantitate the appropriateness of clustering, we calculated silhouette scores for each point using a built-in MATLAB function. Silhouette scores are calculated as

$$F=\frac{b(i)-a(i)}{\max\{a(i),b(i)\}},$$ (3)

where a(i) is the average distance between each point and other points in their own cluster, and b(i) is the average distance between each point and the points in the opposite cluster. We plotted the silhouette score for each point in a silhouette plot. Sensitivity, specificity, accuracy, positive predictive value (PPV), and negative predictive value (NPV) were calculated for each clustering scheme. Sensitivity was calculated as the number of true positives divided by the sum of true positives and false negatives. Specificity was calculated as the true negatives divided by the sum of true negatives and false positives. Accuracy was defined as the sum of true positives and true negatives over the sum of true negatives, false negatives, true positives, and false positives. PPV is the number of true positives over the sum of true and false positives. NPV is the number of true negatives over the sum of true and false negatives. For all above calculations, “positive” refers to tumor and “negative” refers to mammary.

## 3 Results

### 3.1 Mitigating Optical Crosstalk between Bodipy FL C16 and 2-NBDG Fluorescence in Turbid Phantoms

**Fig. 2:**
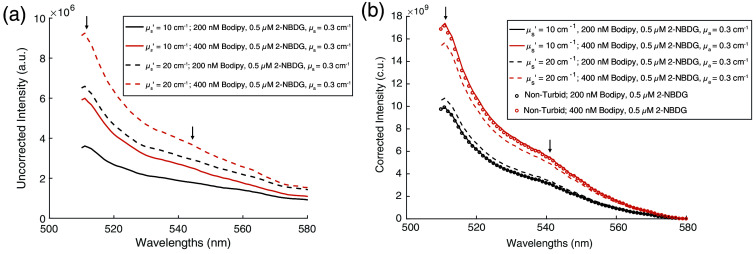
Correction with an inverse Monte Carlo model allows for the accurate extraction of fluorescence in the presence of scatterer and absorber. Two sets of phantoms were constructed containing two fluorophores, non-fluorescent absorber hemoglobin, and scatterer in PBS/BSA. In the first phantom set, the scattering level was set to ${\mu_{s}}^{\prime }=10\;{\mathrm{cm}}^{-1}$ (solid lines). In the second phantom set, the scattering level was set to ${\mu_{s}}^{\prime }=20\;{\mathrm{cm}}^{-1}$ (dashed lines). Both phantom sets contained a fixed $\mu_{a}$ ($0.3\;{\mathrm{cm}}^{-1}$) and 2-NBDG concentration (0.5  μM) and Bodipy FL C16 at two concentrations: 200 nM (black) and 400 nM (red). (a) Measured (uncorrected) spectra from the above phantoms. (b) Spectra from the above phantoms, corrected by inverse Monte Carlo. Black lines represent phantoms containing 200 nM Bodipy FL C16 and red lines represent phantoms containing 400 nM Bodipy FL C16. Dashed lines represent ${\mu_{s}}^{\prime }=20\;{\mathrm{cm}}^{-1}$ and solid lines represent ${\mu_{s}}^{\prime }=10\;{\mathrm{cm}}^{-1}$. Here, we also show non-turbid phantoms, which consist of Bodipy FL C16 (200 nM, black open circles and 400 nM, red open circles) and 2-NBDG (0.5  μM) in PBS/BSA. Arrows point to each fluorophore peak (Bodipy FL C16 = 512 nm, 2-NBDG = 545 nm). In both panels, the reported spectra are mixtures of Bodipy FL C16 and 2-NBDG (no linear unmixing has been applied).

We first sought to show that we can spectrally unmix Bodipy FL C16 and 2-NBDG after correcting for turbidity in a phantom with both an absorber and scatterer. Figure 2 shows two sets of phantoms each containing Bodipy FL C16 and 2-NBDG, a non-fluorescent absorber (hemoglobin), and scatterer (microspheres) in PBS/BSA. In the first phantom set, the scattering level was set to ${\mu_{s}}^{\prime }=10\;{\mathrm{cm}}^{-1}$ (solid lines). In the second phantom set, the scattering level was set to ${\mu_{s}}^{\prime }=20\;{\mathrm{cm}}^{-1}$ (dashed lines). Both phantom sets contained hemoglobin with fixed $\mu_{a}=0.3\;{\mathrm{cm}}^{-1}$, 2-NBDG at a single concentration (0.5  μM), and Bodipy FL C16 at two concentrations (200 nM (black) and 400 nM (red)). Fluorophore concentrations, scattering, and absorption levels in this and the following phantom studies are biologically relevant and based on previous *in vivo* data within the dynamic range of our system.^11^^,^^19^^,^^20^ Figure 2(a) shows uncorrected spectra, in which measured fluorescence intensity varies with scattering level despite the concentration of fluorophore remaining constant. When accounting for scattering levels using an inverse Monte Carlo model [Fig. 2(b)], the corrected fluorescence intensity across wavelengths matches the spectra collected from a non-turbid phantom containing only PBS/BSA and fluorophore (no absorber or scatterer). Without scatterer, the measured fluorescence intensity of the non-turbid phantom was significantly diminished, necessitating a scale factor to compare to the turbid phantom data. The intensity of the non-turbid phantom was scaled by the ratio of the peak of the turbidity-corrected phantom containing 200 nM Bodipy FL C16 and ${\mu_{s}}^{\prime }=20\;{\mathrm{cm}}^{-1}$ to the peak of the non-turbid phantom containing 200 nM Bodipy FL C16, a method established previously.^20^

Bodipy FL C16 and 2-NBDG have peak excitation wavelengths of 502 and 465 nm, respectively, and a peak emission wavelength of 512 and 545 nm, respectively.^18^^,^^27^ Fluorescence emission spectra were acquired from 520 to 600 nm using an excitation wavelength of 488 nm; therefore, we expect optical crosstalk due to spectral bleed-through. We used linear least squares fit to unmix the turbidity-corrected spectrum containing both fluorophores. Our group has previously reported that there is indeed no optical crosstalk between TMRE and either Bodipy FL C16 or 2-NBDG^19^^,^^20^ and is therefore not shown here.

**Fig. 3:**
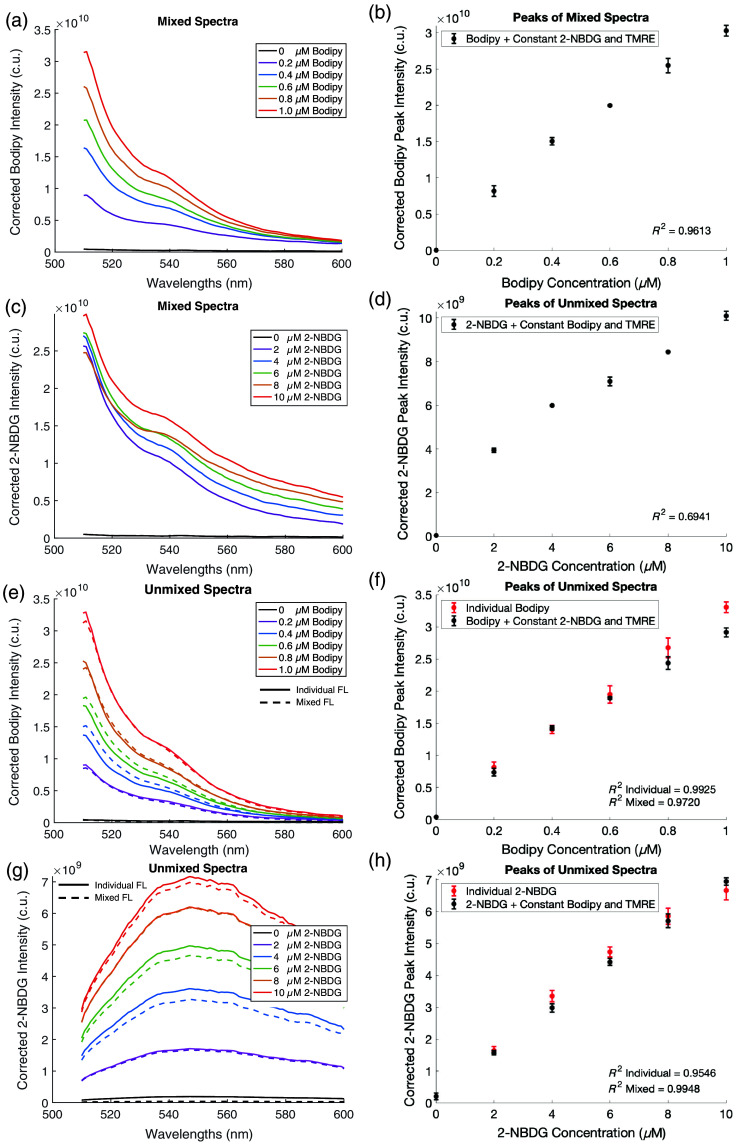
Optical crosstalk between Bodipy FL C16 and 2-NBDG can be mitigated using spectral unmixing; employing both inverse Monte Carlo correction and spectral unmixing yields concordant results between individual preparations of each fluorophore and mixed preparations of Bodipy FL C16, 2-NBDG, and TMRE. Spectral unmixing is necessary due to the overlap in excitation and emission peaks of Bodipy FL C16 (excitation: 505 nm/emission: 512 nm) and 2-NBDG (excitation: 465 nm/emission: 545 nm). We can assume that the combined spectrum is a linear combination of the two individual fluorophore spectra and solve an equation with two unknowns when we have the reference spectrum for each fluorophore. (a) Inverse Monte Carlo corrected, but not linearly unmixed, spectra for mixed phantoms containing Bodipy FL C16 varying between 0 and 1  μM with constant 2-NBDG and TMRE. (b) Peak intensity (512 nm) after inverse Monte Carlo correction, but not linear unmixing. (c) Unmixed Bodipy FL C16 spectra for mixed phantoms containing constant 2-NBDG and constant TMRE (dashed lines) and individual phantoms containing only Bodipy FL C16 (solid lines). (d) Peak intensity (512 nm) after MC correction and unmixing. (e) Inverse Monte Carlo corrected, but not linearly unmixed, spectra for mixed phantoms containing 2-NBDG varying between 0 and 10  μM with constant Bodipy FL C16 and TMRE. (f) Peak intensity (545 nm) after inverse Monte Carlo correction, but not linear unmixing. (g) Unmixed 2-NBDG spectra for mixed phantoms containing constant Bodipy FL C16 and TMRE (dashed lines) and individual phantoms containing only 2-NBDG (solid lines). (h) Peak intensity (545 nm) after inverse Monte Carlo correction and unmixing. All spectra shown are representative spectra from one of three experimental replicates. All peak intensity values are averaged across three experimental replicates; error bars are standard error of the mean.

We sought to demonstrate unmixing of inverse Monte Carlo corrected spectra first in a simple phantom with the fewest number of variables, including fluorophores and scattering spheres only, without the addition of hemoglobin. We show that the sum of the fits for each fluorophore is equivalent to the measured (mixed) spectra, demonstrating complete and accurate unmixing, in Fig. S1 in the Supplementary Material. Figure 3(a) shows the inverse Monte Carlo corrected spectra for a single phantom set containing varying concentrations of Bodipy FL C16 mixed with constant 2-NBDG, TMRE, and scatterer (${\mu_{s}}^{\prime }=10\;{\mathrm{cm}}^{-1}$, these phantoms do not contain hemoglobin). These spectra have not been unmixed. Figure 3(b) shows the peak intensity at 512 nm of these mixed spectra; the $R^{2}$ value is 0.9613. Figure 3(c) shows the spectra after applying linear unmixing of phantoms containing individual Bodipy FL C16 (solid lines) and Bodipy FL C16 mixed with constant 2NBDG and TMRE (dashed lines) and scatterer (${\mu_{s}}^{\prime }=10\;{\mathrm{cm}}^{-1}$). The intensity at the peak Bodipy FL C16 wavelength (512 nm) from the unmixed spectra is shown in Fig. 3(d) for individual Bodipy FL C16 (red dots) and Bodipy FL C16 in a milieu of constant 2-NBDG, TMRE, and scatterer (black dots). Figure 3(d) shows that there is strong concordance between varying concentrations of Bodipy FL C16 in mixed versus individual phantoms. The $R^{2}$ value for the individual Bodipy FL C16 preparation is 0.9925 and the $R^{2}$ value for the mixed Bodipy FL C16 preparation is 0.9720. Figure 3(e) shows the inverse Monte Carlo corrected spectra for phantoms containing 2-NBDG mixed with constant Bodipy FL C16 and TMRE and scatterer (${\mu_{s}}^{\prime }=10\;{\mathrm{cm}}^{-1}$). These spectra are not spectrally unmixed. Figure 3(f) shows the peak intensity at 545 nm of these mixed spectra, with an $R^{2}$ value of 0.6941. The unmixed spectra for 2-NBDG prepared individually (solid lines) and in a mixture of a Bodipy FL C16 and TMRE at constant concentrations and fixed scattering (dashed lines) is shown in Fig. 3(g). After applying linear unmixing, the 2-NBDG peaks (545 nm) increase linearly, as shown in Fig. 3(h), with individual 2-NBDG (red dots) having an $R^{2}$ value of 0.9546 and 2-NBDG in a milieu Bodipy FL C16 and TMRE (black dots) having an $R^{2}$ value of 0.9948. All spectra shown are representative spectra from one of three experimental replicates. All peak intensity values are averaged across three experimental replicates; error bars are the standard error of the mean. From here forward, all reported Bodipy FL C16 and 2-NBDG values have been spectrally unmixed.

**Fig. 4:**
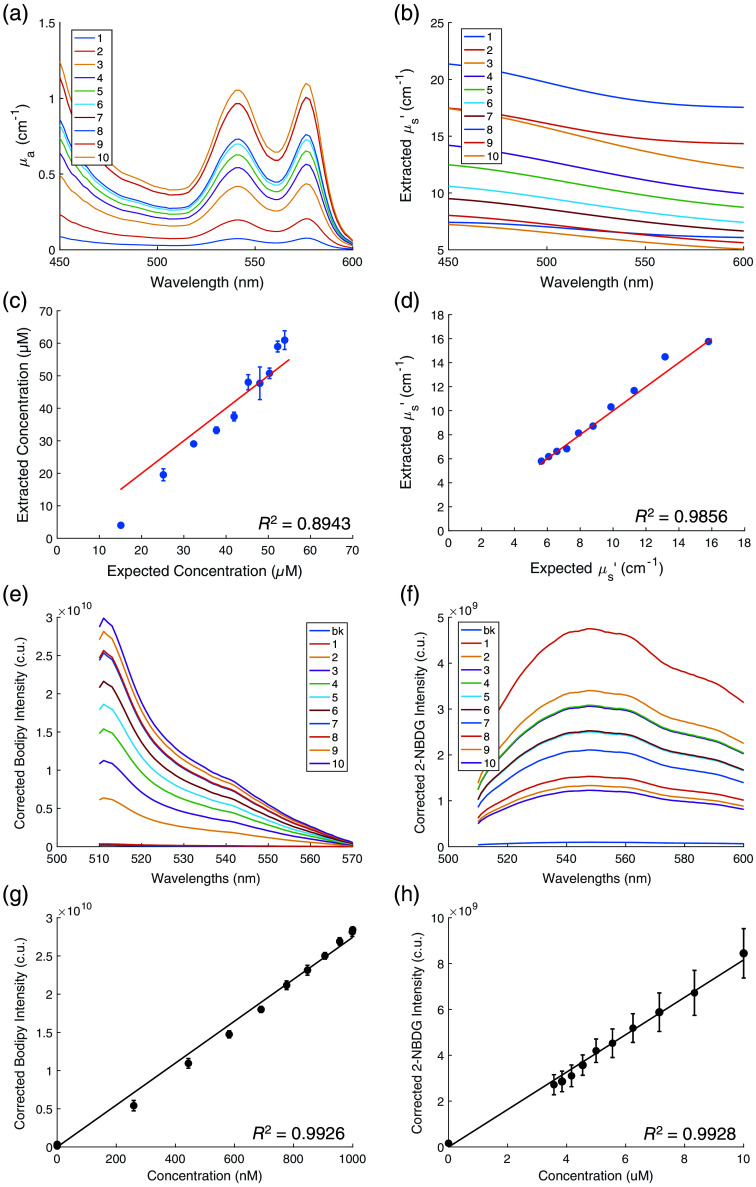
The fluorescence of 2-NBDG and Bodipy FL C16 along with optical properties can be extracted from turbid phantoms. (a) Representative extracted $\mu_{a}$ (${\mathrm{cm}}^{-1}$) and (b) extracted ${\mu_{s}}^{\prime }$ (${\mathrm{cm}}^{-1}$) across wavelengths 450 to 600 nm for each of 10 phantoms. (c) Expected versus extracted hemoglobin concentration (μM); each point represents the average of three replicates and error bars are SE (${\mathrm{cm}}^{-1}$, $R^{2}=0.8943$). Expected hemoglobin concentration is calculated using absorbance measured by UV-VIS while extracted hemoglobin concentration is calculated using $\mu_{a}$ extracted by inverse Monte Carlo. (d) Expected versus extracted ${\mu_{s}}^{\prime }$; each point represents the average of three replicates and error bars are SE (${\mu_{s}}^{\prime }$, ${\mathrm{cm}}^{-1}$, $R^{\prime }=0.9856$). Error bars are present though hard to visualize due to their size; a table of SE values can be found in Table S1 in the Supplementary Material. The red lines in (c) and (d) represent y=x. Representative corrected (e) Bodipy FL C16 and (f) 2-NBDG spectra for all 10 phantoms. Extracted fluorescence signal versus concentration for (g) Bodipy FL C16 ($R^{2}=0.9926$) and (h) 2-NBDG ($R^{2}=0.9928$). Points in (g) and (h) represent the average of three replicates and error bars are SE. The black lines in (g) and (h) represent the line of best fit line with the y intercept through 0. All values are MC corrected; intensity is reported in corrected units (c.u.). Bodipy FL C16 and 2-NBDG spectra have been spectrally unmixed.

Finally, we sought to show that the inverse Monte Carlo is capable of extracting Bodipy FL C16 and 2-NBDG fluorescence intensity as well as optical properties in a more complex, tissue-mimicking phantom set in which all components are varied across a range of $\mu_{a}$, ${\mu_{s}}^{\prime }$, and fluorophore concentrations comparable to what we expect to see *in vivo*. We thus constructed a series of 10 phantoms in which 2-NBDG concentration and ${\mu_{s}}^{\prime }$ decreased as Bodipy FL C16 concentration and $\mu_{a}$ increased (Table 3). The choice for Bodipy FL C16 to increase and 2-NBDG to decrease mimics the known *in vivo* uptake kinetics of each fluorophore over the course of 60 min after injection into a live animal.^11^^,^^19^^,^^20^ This experiment involved repeated measurements (three replicates) and we report representative spectra [Figs. 4(a), 4(b), 4(e), and 4(f)] and extracted values averaged across three replicates [Figs. 4(c), 4(d), 4(g), and 4(h)].

Figures 4(a) and 4(b) show extracted $\mu_{a}$ and ${\mu_{s}}^{\prime }$ spectra (from one representative experimental replicate), respectively, for each of the ten phantoms. Figure 4(c) shows the average extracted versus expected hemoglobin concentration across three replicates with $R^{2}=0.8943$. Figure 4(d) shows the average extracted versus expected ${\mu_{s}}^{\prime }$ across three replicates with $R^{2}=0.9856$. Figures 4(e) and 4(f) show inverse Monte Carlo corrected and spectrally unmixed Bodipy FL C16 and 2-NBDG spectra, respectively, for all 10 phantoms and Figs. 4(g) and 4(h) show corrected fluorescence peak values for Bodipy FL C16 (512 nm) and 2-NBDG (545 nm), respectively. As expected, both Bodipy FL C16 and 2-NBDG fluorescence increase linearly with concentration with $R^{2}$ values of 0.9926 and 0.9928, respectively. The solid red line in Figs. 4(c) and 4(d) is y=x or perfect agreement between expected and extracted. The solid black line in Figs. 4(g) and 4(h) is the line of best fit for concentration versus intensity.

### 3.2 Bodipy FL C16 and 2-NBDG Do Not Interfere with Each Other Biologically in a Staggered Injection Scheme

**Fig. 5:**
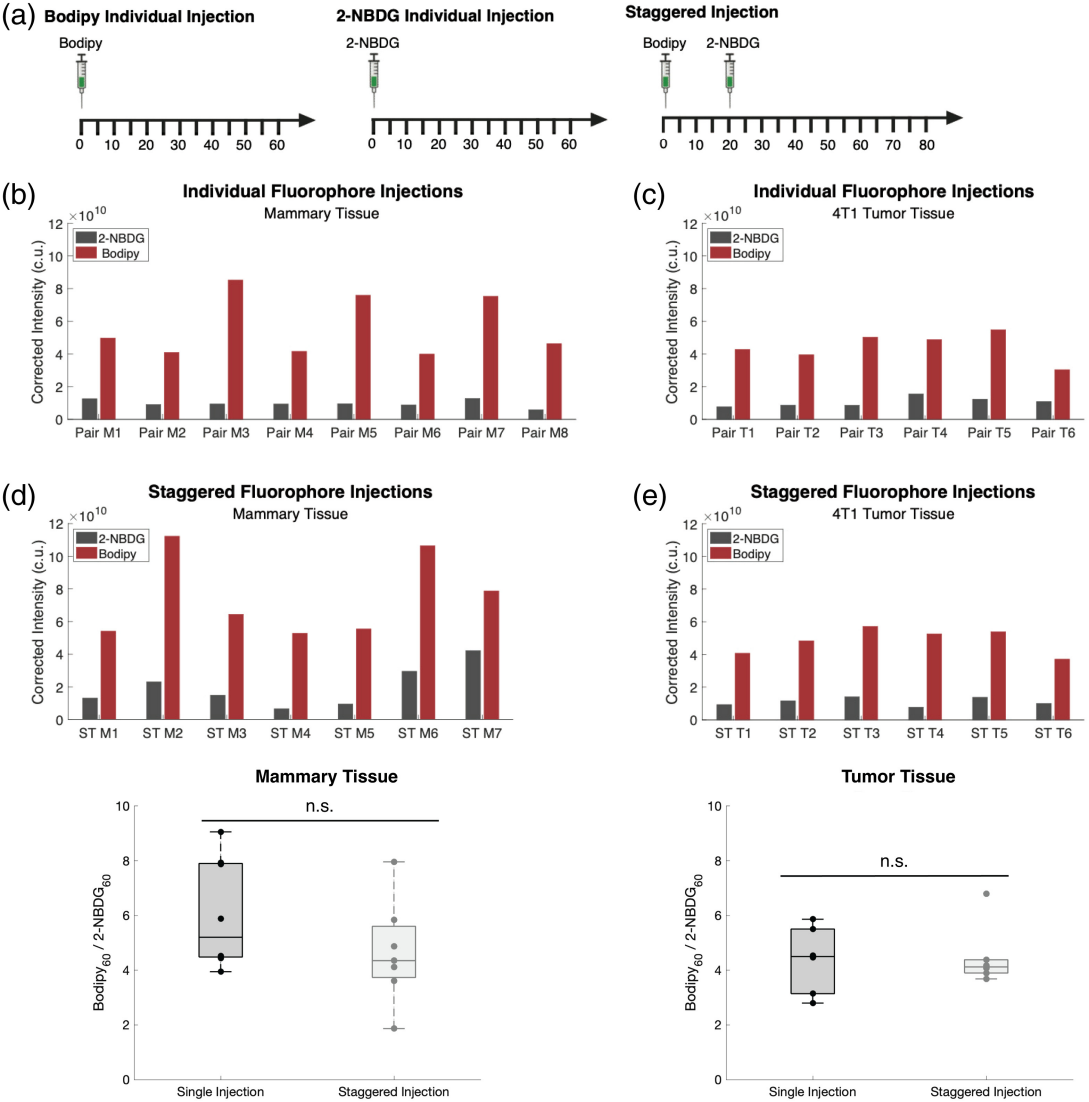
There is no biological crosstalk between Bodipy FL C16 and 2-NBDG. (a) Schematic of the two injection schemes reported here. In the staggered injection scheme, Bodipy FL C16 (100  μL, 200  μM) is injected first followed by 2-NBDG (100  μL, 6 mM) 20 min later, and both are measured for 60 min post-injection. In the single injection scheme, either Bodipy FL C16 or 2-NBDG is injected and measured for 60 min post-injection. (b) $2{\mathrm{NBDG}}_{60}$ (black) and ${\text{Bodipy}}_{60}$ (red) intensity for pairs of mice receiving individual injections of Bodipy FL C16 or 2-NBDG in mammary tissue. (c) $2\text{-}{\mathrm{NBDG}}_{60}$ (black) and ${\text{Bodipy}}_{60}$ (red) intensity for pairs of mice receiving individual injections of Bodipy FL C16 or 2-NBDG in 4T1 tumor tissue. In (b), (c), pairs are labeled as “Pair M1,” etc. for mammary tissue and “Pair T1” etc. for tumor tissue. (d) $2\text{-}{\mathrm{NBDG}}_{60}$ (black) and ${\text{Bodipy}}_{60}$ (red) intensity for mice receiving a staggered injection of Bodipy FL C16 and 2-NBDG in mammary tissue. (e) $2\text{-}{\mathrm{NBDG}}_{60}$ (black) and ${\text{Bodipy}}_{60}$ (red) intensity mice receiving a staggered injection of Bodipy FL C16 and 2-NBDG in 4T1 tumor tissue. In (d), (e), mice are labeled as “ST M1” etc. for staggered injection in mammary tissue and “ST T1” etc. for staggered injection in tumor tissue. In mammary tissue (f) and 4T1 tumor tissue (g), the ratio of ${\text{Bodipy}}_{60}/2\text{-}{\mathrm{NBDG}}_{60}$ for each pair of mice (for single injections) is not significantly different than from the ratio of ${\text{Bodipy}}_{60}/2\text{-}{\mathrm{NBDG}}_{60}$ for mice receiving the staggered injection (p=n.s. by Wilcoxon rank-sum test). Dots represent individual mice or mouse pairs.

Next, we sought to demonstrate that Bodipy FL C16 and 2-NBDG do not react biologically in an *in vivo* system. Previous work by our group has demonstrated that biological crosstalk between TMRE and 2-NBDG is eliminated if the delivery of the two fluorophores is temporally spaced such that 2-NBDG is injected 20 min after TMRE injection; this is referred to as a near-simultaneous injection scheme.^14^ Further work has demonstrated that a combined injection of Bodipy FL C16 and TMRE is suitable to accurately report on both endpoints.^20^ Ultimately, we aim to integrate Bodipy FL C16 into the near-simultaneous injection scheme validated previously. Therefore, showing that Bodipy FL C16 followed by 2-NBDG after 20 min (staggered) can faithfully recapitulate the uptake of Bodipy FL C16 or 2-NBDG by themselves sets the stage for a successful concurrent three fluorophore injection. The individual and staggered injection schemes are shown in Fig. 5(a).

Both healthy and tumor-bearing female BALB/c mice were used to investigate biological crosstalk *in vivo*. Healthy mammary and tumor tissue types are not only both relevant to preclinical studies but also represent distinct vascular and metabolic environments in which to measure our endpoints. As both Bodipy FL C16 and 2-NBDG have been previously validated as measures of lipid and glucose uptake respectively *in vivo*, the goal was to demonstrate concordance between single injections and staggered injections of these two fluorophores.

Each fluorophore was delivered retro-orbitally; the 60-min post-injection timepoint for each fluorophore was used for all analyses. We injected each fluorophore individually into pairs of animals, and Fig. 5 shows ${\text{Bodipy}}_{60}$ and $2\text{-}{\mathrm{NBDG}}_{60}$ for each animal pair receiving individual injections [Figs. 5(b) and 5(c)] and the staggered injection scheme [Figs. 5(d) and 5(e)] for mammary and tumor tissue, respectively. A Wilcoxon rank-sum test reveals that there is no significant difference in the ratio of ${\text{Bodipy}}_{60}$ to $2\text{-}{\mathrm{NBDG}}_{60}$ for the individual injection scheme versus the staggered injection scheme [Figs. 5(f) and 5(g)] for mammary and tumor tissue, respectively.

### 3.3 Bodipy FL C16, 2-NBDG, and TMRE Can Be Combined in a Concurrent Injection Scheme in a Single Animal to Improve Discrimination Between Tumor Bearing and Non-Tumor Bearing Mammary Tissues

Previous reports from our group show that Bodipy FL C16 and TMRE can be combined in a single injection^20^ and that TMRE and 2-NBDG can be administered near-simultaneously in two injections without biological crosstalk.^14^^,^^19^ Further Figs. 3 and 4 demonstrate that inverse Monte Carlo correction and spectral unmixing mitigates optical crosstalk between Bodipy FL C16 and 2-NBDG. Figure 5 indicates that Bodipy FL C16 and 2-NBDG delivered in a staggered injection scheme do not show biological crosstalk. Therefore, we developed a concurrent injection scheme in which Bodipy FL C16 was co-delivered with TMRE in a single injection followed by 2-NBDG delivery after a 20-min delay.

**Fig. 6:**
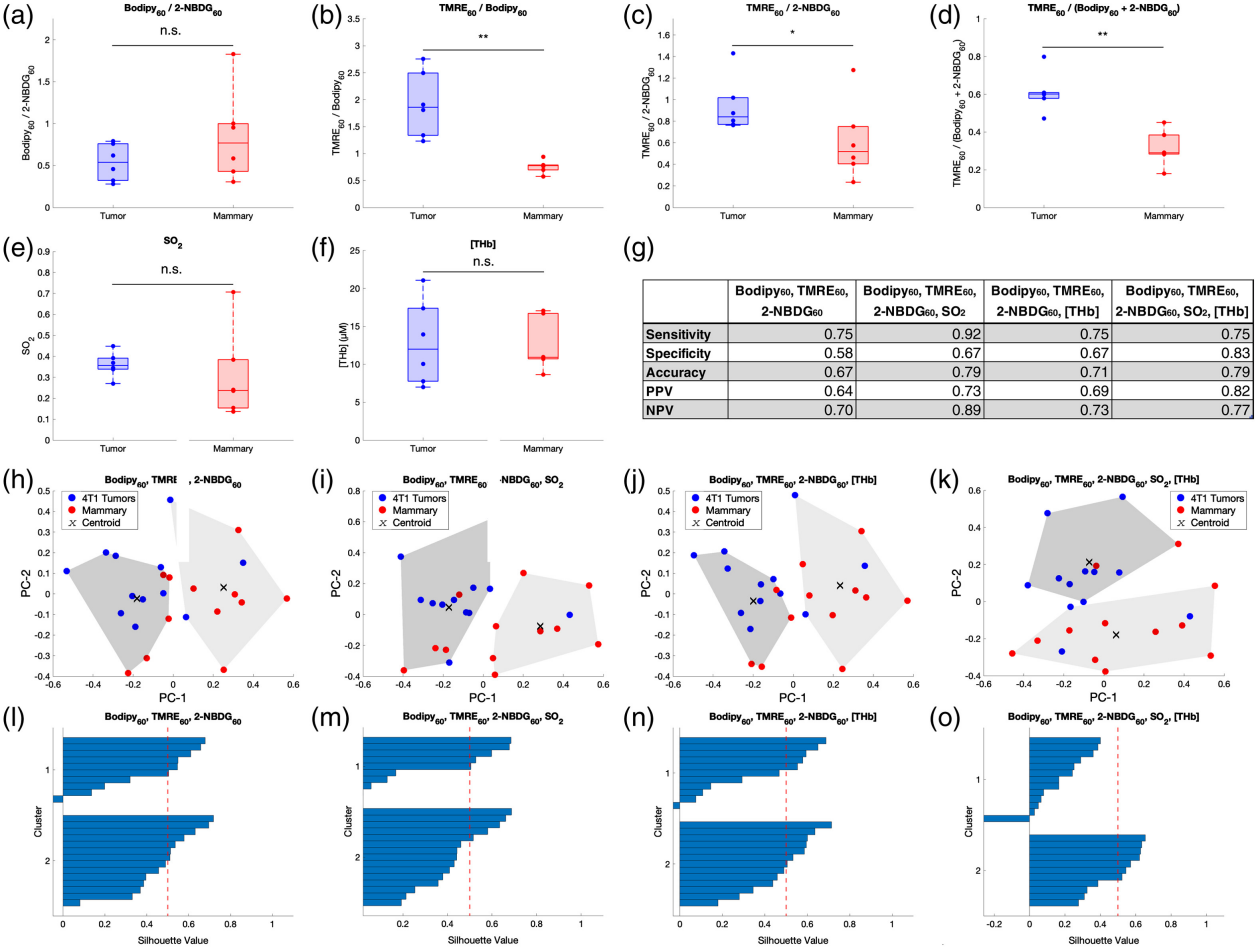
Integrating five endpoints provides additional information with which to differentiate tumor from healthy mammary tissue. (a) The ratio of ${\text{Bodipy}}_{60}/2\text{-}{\mathrm{NBDG}}_{60}$ for tumor and mammary tissue (p=n.s.). (b) ${\mathrm{TMRE}}_{60}/{\text{Bodipy}}_{60}$ for tumor and mammary tissue (p=0.01). (c) The ratio of ${\mathrm{TMRE}}_{60}/2\text{-}{\mathrm{NBDG}}_{60}$ for tumor and mammary tissue (p<0.01). (c) The ratio of ${\mathrm{TMRE}}_{60}/2\text{-}{\mathrm{NBDG}}_{60}$ for tumor and mammary tissue (p<0.05). (d) The ratio of ${\mathrm{TMRE}}_{60}/({\text{Bodipy}}_{60}+2\text{-}{\mathrm{NBDG}}_{60})$ for tumor and mammary tissue (p<0.01). (e) ${\mathrm{SO}}_{2}$ for tumor and mammary tissue (p=n.s.). (f) [THb] (μM) for tumor and mammary tissue (p=n.s.). All comparisons are tested by a Wilcoxon rank-sum test. (g) The sensitivity, specificity, accuracy, PPV, and NPV for each combination. PCA was performed and the first three principal components were plotted. Clustering was performed on the first three principal components (accounting for between 77% and 100% of variance). Projections onto the PC1 and PC2 axes are displayed for k-means clustering of the principal components for (h) three metabolic endpoints: ${\text{Bodipy}}_{60}$, ${\mathrm{TMRE}}_{60}$, $2\text{-}{\mathrm{NBDG}}_{60}$, (i) four endpoints: ${\mathrm{Bodipy}}_{60}$, ${\mathrm{TMRE}}_{60}$, $2\text{-}{\mathrm{NBDG}}_{60}$, and ${\mathrm{SO}}_{2}$, (j) four endpoints: ${\text{Bodipy}}_{60}$, ${\mathrm{TMRE}}_{60}$, $2\text{-}{\mathrm{NBDG}}_{60}$, and [THb], and (k) five endpoints: ${\text{Bodipy}}_{60}$, ${\mathrm{TMRE}}_{60}$, $2\text{-}{\mathrm{NBDG}}_{60}$, [THb], and ${\mathrm{SO}}_{2}$. Blue dots represent datapoints measured from tumor tissue and red dots represent datapoints measured from mammary tissue. Light and dark gray boundaries represent the two clusters determined by k-means, and black crosses represent the centroids. The first three principal components explain (h) 100% of variance, (i) 85.06% of variance, (j) 83.67% of variance, and (k) 77.40% of variance. (l)–(o) Silhouette plots for each cluster analysis. The red dashed line represents a silhouette score of 0.5, a score generally considered to represent a point being well-matched to its own cluster.

Next, we sought to explore the biological utility of the information gained from the three metabolic endpoints and two vascular endpoints by measuring our fluorophores in 4T1 tumors and healthy mammary tissue. Figures 6(a)–6(f) show the ratios ${\text{Bodipy}}_{60}/2\text{-}{\mathrm{NBDG}}_{60}$, ${\mathrm{TMRE}}_{60}/{\text{Bodipy}}_{60}$, ${\mathrm{TMRE}}_{60}/2\text{-}{\mathrm{NBDG}}_{60}$, ${\mathrm{TMRE}}_{60}$/the sum of both substrates (${\text{Bodipy}}_{60}+2\text{-}{\mathrm{NBDG}}_{60}$), and finally ${\mathrm{SO}}_{2}$ and [THb] from the concurrent injection scheme. A Wilcoxon rank-sum test reveals that the ratio of ${\mathrm{TMRE}}_{60}/{\text{Bodipy}}_{60}$, ${\mathrm{TMRE}}_{60}/2\text{-}{\mathrm{NBDG}}_{60}$, and ${\mathrm{TMRE}}_{60}/{\text{Bodipy}}_{60}+2\text{-}{\mathrm{NBDG}}_{60}$ are significantly increased in tumor compared to normal tissue. [THb] and ${\mathrm{SO}}_{2}$ do not show statistically significant differences between tumor and mammary tissue (p=n.s. for all comparisons). The ability to measure all biological endpoints together allows for reduction of inter-tumor variability. Therefore, we can construct a meaningful ratio by dividing ${\mathrm{TMRE}}_{60}$ by the sum of both ${\text{Bodipy}}_{60}$ and $2\text{-}{\mathrm{NBDG}}_{60}$.

Although ${\mathrm{SO}}_{2}$ and [THb] did not show statistically significant differences between tumor and mammary tissue, clustering analysis showed that combining metabolic and vascular endpoints outperformed the use of metabolic endpoints alone to separate tumor versus mammary tissue. We used PCA to reduce the dimensionality of our dataset, and k-means clustering of the first three PCs for different combinations of our five endpoints. Figure 6(g) shows the sensitivity, specificity, accuracy, PPV, and NPV for each combination indicating the benefits of using all five endpoints. Figures 6(h) and 6(k) show k-means clustering of the first three PCs for different combinations of our five endpoints: ${\text{Bodipy}}_{60}$, ${\mathrm{TMRE}}_{60}$, $2\text{-}{\mathrm{NBDG}}_{6}$; ${\text{Bodipy}}_{60}$, ${\mathrm{TMRE}}_{60}$, $2\text{-}{\mathrm{NBDG}}_{60}$, and ${\mathrm{SO}}_{2}$; ${\text{Bodipy}}_{60}$, ${\mathrm{TMRE}}_{60}$, $2{\mathrm{NBDG}}_{60}$, and [THb]; and ${\text{Bodipy}}_{60}$, ${\mathrm{TMRE}}_{60}$, $2\text{-}{\mathrm{NBDG}}_{60}$, ${\mathrm{SO}}_{2}$, and [THb], respectively. Figures 6(l)–6(o) show the silhouette plots for each combination, respectively, showing the appropriateness of clustering by quantitating the average distance between each point and other points in their own cluster and the average distance between each point and the points in the opposite cluster.

## 4 Discussion and Conclusion

In this study, we demonstrate the ability to make time-matched measurements of glucose and fatty acid uptake alongside mitochondrial activity in a single timepoint in a single animal. We can also track vascular properties ${\mathrm{SO}}_{2}$ and [THb], which are relevant to nutrient delivery and oxygenation of the tissue, variables affecting metabolism. This work builds on previous studies conducted by our group, which demonstrated that Bodipy FL C16 and TMRE can be simultaneously delivered with neither optical nor biological crosstalk.^20^ While the TMRE spectrum does not overlap with that of Bodipy FL C16, the excitation and emission spectra of Bodipy FL C16 and 2-NBDG overlap. Therefore, addressing optical crosstalk between the two fluorophores will allow us to combine all three fluorophores in a concurrent injection scheme to track the three metabolic endpoints and the two vascular endpoints *in vivo*.

In previous publications, we have robustly validated each fluorophore using complementary gold standard biological assays.^3^^,^^8^^,^^11^^,^^14^^–^^20^ We have shown in various models, including oncogene switchable models comparing oncogene driven versus oncogene downregulated tumor tissue and tumor tissue following treatment, that our optical toolkit is in concordance with gold-standard techniques (RNAseq and metabolomics) and complementary biological assays (IHC).^3^^,^^8^^,^^11^^,^^36^ Therefore, concordance between concurrent versus individual measurements is sufficient to demonstrate the utility of the combination over individual measurements.

While dysregulated metabolism drives breast cancer aggressiveness and progression, a focus on glucose metabolism alone is insufficient. The addition of Bodipy FL C16 to the metabolic repertoire allowing for monitoring of fatty acid uptake is essential to understanding tumor resistance to treatment. Tumor-surrounding adipocytes release free fatty acids for cancer cells to uptake, which in turn increases tumor aggressiveness and invasiveness.^37^ The breast tissue is replete with adipocytes, and it has increasingly been shown pre-clinically and clinically that fats play a complex role in signaling and sustaining metabolic switches that drive breast cancer progression.^37^ Understanding whether fatty acids are being oxidized via fatty acid β-oxidation to fuel OXPHOS will provide a more holistic picture of cellular energetics. Therefore, incorporating Bodipy FL C16 alongside TMRE and 2-NBDG to our injection scheme has the potential to expand our understanding of the substrates that fuel OXPHOS in aggressive breast cancers. We set out to demonstrate increased utility of additional endpoints collected concurrently using a system capable of longitudinal and non-invasive *in vivo* measurements, and future studies can be designed to answer biological questions regarding fatty acid uptake in TNBC.

Our inverse Monte Carlo algorithm corrects for fluorescence aberrations caused by tissue optical properties.^38^ Figure 2(a) demonstrates that varying scattering levels affect the fluorophore measurement; in Fig. 2(b), the effect is corrected by the inverse Monte Carlo algorithm. We have demonstrated this previously with fluorophores TMRE, 2-NBDG, and Bodipy FL C16 individually.^20^ In Fig. 2, we expanded upon previous studies by demonstrating this in a combined phantom containing both 2-NBDG and Bodipy FL C16: the two fluorophores that have overlapping excitation and emission spectra.

In Fig. 3, we observe that 2-NBDG peak fluorescence intensity does not increase linearly in the presence of Bodipy FL C16 before spectral unmixing ($R^{2}=0.6941$), although the linear relationship is recovered after spectral unmixing ($R^{2}=0.9635$). Conversely, Bodipy FL C16 maintains a linear increase in fluorescence intensity with increasing concentration, even in the presence of 2-NBDG [Fig. 3(b), $R^{2}$ value is 0.9790], likely due to the difference in absolute intensity between Bodipy FL C16 and 2-NBDG, with Bodipy FL C16 having a higher quantum yield than 2-NBDG. The effect of 2-NBDG fluorescence intensity on the peak of Bodipy FL C16 is negligible, whereas the intensity of Bodipy FL C16 is non-negligible compared to 2-NBDG signal before unmixing.

In Fig. 4, we show Bodipy FL C16 and 2-NBDG, the two fluorophores yet to be combined, in a more complex model that mimics tissue by varying concentrations of fluorophore and tissue properties spanning a range relevant to what we see in tissue. This demonstrates our ability to extract measurements of Bodipy FL C16 and 2-NBDG along with $\mu_{a}$, ${\mu_{s}}^{\prime }$ from a model closely mimicking tissue, paving the way for testing *in vivo*. In our most recent publication, we constructed a tissue-mimicking phantom with the same range of Bodipy FL C16 concentration, $\mu_{a}$, ${\mu_{s}}^{\prime }$ (with TMRE instead of 2-NBDG); here we conducted similar experiments for these endpoints.^20^ Of note, the $R^{2}$ value for the expected versus extracted hemoglobin concentration [Fig. 4(c)] is less than the $R^{2}$ values for the expected versus extracted ${\mu_{s}}^{\prime }$ and corrected fluorophore intensities ($R^{2}=0.8943$ versus $R^{2}=0.9856$, $R^{2}=0.9926$, and $R^{2}=0.9928$, respectively). This may be explained by the fact that we used publicly available reference spectra for oxy- and deoxyhemoglobin. Phantoms prepared with commercially available hemoglobin may contain varying amounts of methemoglobin not accounted for in publicly available reference spectra.^39^ We do not expect that this is a problem for *in vivo* studies.

Having validated our spectral unmixing approach to mitigate optical crosstalk, our next experiment addressed biological crosstalk. While our data from Figs. 3 and 4 suggest that Bodipy FL C16 can be measured simultaneously, we chose to space them temporally in a staggered injection to integrate Bodipy FL C16 into the established injection scheme by injecting TMRE and Bodipy FL C16 simultaneously followed by 2-NBDG after a delay of 20 min. Our analysis indicates that the Bodipy FL C16 to 2-NBDG ratio when combined in a single animal reflects what we measure in individual injections of each fluorophore in separate animals (Fig. 5). Thus, we conclude that the administration of two fluorophores in a single animal does not affect our ability to measure the endpoint of each.

Having comprehensively shown that our fluorophores can be combined without biological crosstalk, in Fig. 6, we demonstrate the utility of our concurrent injection scheme in providing additional biological information necessary to differentiate tumor and normal tissues. Specifically, we sought to demonstrate the utility of our concurrent injection scheme by distinguishing tumor from normal tissue first using ratios of our metabolic endpoints and then clustering of all five of our metabolic and vascular endpoints. We report significant differences in tumor compared to normal tissue when reporting ratios of fluorophores (${\mathrm{TMRE}}_{60}/{\text{Bodipy}}_{60}$, ${\mathrm{TMRE}}_{60}/2\text{-}{\mathrm{NBDG}}_{60}$, and ${\mathrm{TMRE}}_{60}/({\text{Bodipy}}_{60}+2\text{-}{\mathrm{NBDG}}_{60})$. We also report higher sensitivity, specificity, accuracy, PPV, and NPV when using combinations of metabolic and vascular endpoints as compared to only metabolic endpoints.

Using optical fluorescence spectroscopy to capture the intensity of Bodipy FL C16 and 2-NBDG across 450 to 600 nm allowed us to leverage the unique spectral shape of each fluorophore for accurate spectral unmixing. Another inherent advantage of optical spectroscopy is that it can monitor solid tumors without the need for any additional modifications to the animal, such as implantation of window chambers. This makes spectroscopy well-suited for longitudinal monitoring; fluorophores clear from the circulation after 48 h, enabling metabolic measurements every 2 days. Further, assuming a 5×5  mm or larger tumor, it is feasible to perform multiple spectroscopy measurements on a few different regions of the palpable tumor surface.

Considering the well documented heterogeneity of tumors, a point measurement on the surface of the tumor may fail to represent the entire tumor. Therefore, our spectroscopic method is intended to complement other methods to characterize tumor metabolism. While spectroscopy enables multi-parametric measurements of various endpoints taken as a single point measurement, optical imaging and in particular, intravital microscopy can capture the spatial heterogeneity of these same endpoints, the timing of which can be guided by spectroscopic monitoring. Further, spectroscopic monitoring could provide insight into time points at which tissues can be harvested for molecular assays to study mechanisms underlying metabolic reprogramming. The spectroscopic endpoints can also be synergistic with other modalities, such as magnetic resonance imaging (MRI) and magnetic resonance spectroscopy (MRS), techniques that use endogenous contrast to measure complementary metabolites in the millimolar range *in vivo*.^40^

Previous work in our lab has validated the use of each fluorophore discussed in this work, Bodipy FL C16, TMRE, and 2-NBDG, for wide-field fluorescence microscopy.^11^^,^^14^^,^^16^^,^^17^ Our spectroscopic method can be used to design concurrent and sequential delivery schemes efficiently, which can then be leveraged for multi-parametric microscopy. The metabolic endpoints captured in future imaging studies can also be related to vascular features, such as micro-vessel density, tortuosity, and vessel diameter to understand the influence of the unbalanced blood supply to nutrient preferences of the tumor.

Multi-parametric microscopy and spectroscopy endpoints can play important roles as functional biomarkers of treatment resistance. Breast cancer pathologic complete response at the time of surgery following treatment can be as low as 35%.^22^ Resistance occurs when even a small group of cancer cells have molecular or phenotypic changes rendering them insensitive to a particular drug. Testing different drugs and waiting for tumors to respond is inherently inefficient, but patient-derived organoids (PDOs) and patient-derived xenografts (PDXs), which maintain clinical signatures of resistance from the tumor of origin, can overcome these challenges by providing a testbed to evaluate recurrence risk and evaluate therapeutic strategies or identify new ones.^41^ Our metabolic toolkit, with the ability to track metabolic changes in PDOs and PDXs, is poised to efficiently test treatment strategies that can inform drug selection for an individual patient by utilizing PDOs via multi-parametric microscopy and characterization of long-term outcomes of promising treatments in PDX models with multi-parametric spectroscopy.

The role of PDOs and PDX is particularly important in adaptive clinical trials (ACTs). ACTs increase the efficiency of randomized clinical trials by allowing intervention arms to be added to or removed from the platform or for patients to switch from one arm to another if the experimental treatment that they are on proves ineffective.^42^ The central advantage of adaptive design is the ability to modify study design elements and treatment alternatives based on interim data analyses,^42^ rather than waiting months or years to determine if the treatment was successful. Spectroscopic measurements of PDX models and microscopy of PDOs can be leveraged as part of the clinical pipeline to track treatment success or adapt treatment as needed in real time during an ACT. Taken together, the ability to monitor metabolism in PDX and PDO models of human breast cancer expands upon the clinical toolbox for evaluation of treatment efficacy.

## Supplementary Material

10.1117/1.BIOS.1.2.025002.s01

## Data Availability

Data may be accessed at the following publicly available online repository: https://doi.org/10.7924/r4vx0k33z. All data can be made available upon request to the corresponding authors.
