# Supplementary material for: In vivo spectroscopy to concurrently characterize five metabolic and vascular endpoints relevant to aggressive breast cancer
Source: Biophotonics Discov. 2024 Jul 17;1(2):025002. doi: 10.1117/1.BIOS.1.2.025002 (PMC11970916; doi:10.1117/1.BIOS.1.2.025002)
Supplement: Supplementary file 1 [file BIOS_001_025002_SD001.docx]

Supplementary Fig. S1. The linear least squares fit algorithm accurately estimates the Bodipy FL C16 and 2-NBDG fits; this is shown by comparing the total of the fits to the collected mixed spectra. The sum of Bodipy FL C16 and 2-NBDG fits resulting from linear unmixing (solid lines), and the Monte Carlo corrected mixed spectra (dashed lines) are equivalent for (a) phantoms where 2-NBDG is held constant and Bodipy FL C16 is added linearly from 0-1 µM and (b) for phantoms where Bodipy FL C16 is held constant and 2-NBDG FL C16 is added linearly from 0-10 µM.

Supplementary Table S1. Standard error for each phantom reported in Fig. 4.


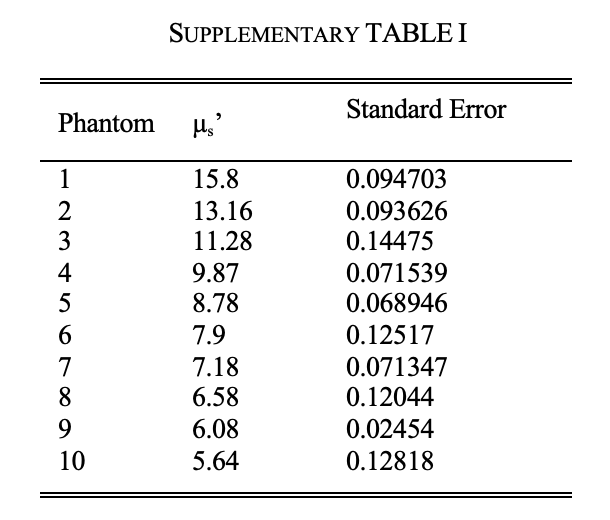

Supplementary Fig. S2. (a) The sensitivity, specificity, accuracy, PPV and NPV for each combination when dimensions are reduced using UMAP and k-means clustering is performed. (b-e) show the UMAP space of (b) three metabolic endpoints: Bodipy_60_, TMRE_60_, 2-NBDG_60_, (c) four endpoints: Bodipy_60_, TMRE_60_, 2-NBDG_60_, and SO_2_, (d) four endpoints: Bodipy_60_, TMRE_60_, 2-NBDG_60_, and [THb], and (e) five endpoints: Bodipy_60_, TMRE_60_, 2-NBDG_60_, [THb], and SO_2_. Blue dots represent datapoints measured from tumor tissue and red dots represent datapoints measured from normal mammary tissue. Light and dark grey boundaries represent the two clusters determined by k-means clustering, and black crosses represent the centroids. (f-i) show silhouette plots for each cluster analysis. The red dashed line represents a silhouette score of 0.5, a score generally considered to represent a point being well-matched to its own cluster.
